# Assessment of knowledge, practices, and barriers to pharmacovigilance among nurses at a teaching hospital, Ghana: a cross‑sectional study

**DOI:** 10.1186/s12912-022-00965-4

**Published:** 2022-08-30

**Authors:** Paa Kofi Tawiah Adu-Gyamfi, Kwesi Boadu Mensah, Joseph Ocansey, Aliu Moomin, Bright Owusu Danso, Frank Agyapong, Reginald Arthur-Mensah Jnr

**Affiliations:** 1 Department of Nursing and Midwifery, Faculty of Health and Allied Sciences, Pentecost University Accra, Accra, Ghana; 2 Department of Pharmacology, Faculty of Pharmacy and Pharmaceutical Sciences, College of Health Science, Kwame Nkrumah University of Science and Technology, Kumasi, Ghana; 3 School of Pharmacy and Life Sciences, Robert Gordon University, Aberdeen, UK

**Keywords:** Pharmacovigilance, Adverse drug reaction, Adverse drug reaction reporting systems, Nurses

## Abstract

**Background:**

Pharmacovigilance may be defined as the continuous monitoring of the reaction between a drug agent or combination of drugs a patient took and steps taken to prevent any associated risk. Clinical trials conducted before drug approval cannot uncover every aspect of the health hazards of approved drugs. People with carefully selected characteristics are monitored for the safety and efficacy of the drug; hence, common adverse drug reactions (ADRs) following proper use of the medication can be detected. This calls for continuous monitoring of drugs to report any undocumented ADRs during the clinical trial. The study aimed to assess the knowledge, practice, and barriers to pharmacovigilance among nurses at a teaching hospital.

**Methods:**

The study was a descriptive cross-sectional study, and a stratified sampling technique was used to select 125 nurses within the three units: medical, surgical, and pediatric wards. A structured questionnaire was developed and used for data collection based on the study's objectives and reviewed literature.

**Results:**

The majority (67.2%) of the respondents were females, and 32.8% were males. Most (71.2%) of the nurses had low knowledge of ADR reporting procedures. Also, 84.8% of the nurses knew the purpose of reporting ADRs. The purpose of ADR reporting, as perceived by respondents, was to identify safe drugs (80.8%) and calculate the incidence of ADR (75.2%). Additionally, among the nurses who reported having nursed a patient with ADRs, 52.54% stated they reported the case, while 47.46% did not report it. The most cited reason for not reporting ADRs was that nurses considered the reaction normal and commonly associated with that medicine (35.7%). In comparison, 28.5% of the nurses said they did not know they were supposed to report the adverse drug reaction. There was no statistically significant difference between ranks of nurses, ward, attending in-service training, and pharmacovigilance practice.

**Conclusion:**

In conclusion, nurses in this study had inadequate knowledge of pharmacovigilance and its reporting procedure. The study found that most nurses fear that reporting ADRs may be wrong because most of the nurses in the study did not have any form of pharmacovigilance training.

## Background

Pharmacovigilance (PV) is the science and activities relating to identifying, understanding, assessing, and preventing risks associated with drugs. One of the most effective ways of monitoring the effective use of drugs in a population is spontaneous adverse drug reporting (SADR) [1]. No drug is harmless and free from adverse effects. There are two major effects of every drug: therapeutic and adverse effects. Anyone using drugs can experience some form of adverse reaction (ADR) [2]. Conducting a pharmacovigilance assessment is one of the most effective ways of monitoring the adverse effects of drug use on a population [3]. Pharmaceutical companies usually conduct a clinical trial of drugs to determine the therapeutic and harmful effects before the drugs are made available on the market for use [4].

A clinical trial conducted before drug approval does not show every aspect of related adverse effects of the approved drug. Prolonged use of medication can help detect common ADRs [5]. This calls for continuous monitoring of drugs to report any undocumented ADRs during the clinical trial.

The prevalence of ADRs is increasing globally and has become one of the leading causes of morbidity and mortality in developed countries [6]. ADRs also prolong the hospital admissions of a patient. A prospective study showed that the average number of days spent in hospitals was eight days for patients without ADRs compared to 20 days for patients with ADRs [7].

ADRs are a severe problem in health care delivery, particularly during the management of chronic diseases. ADRs are a primary reason for therapy change and drug withdrawal from the market and affect adherence to treatment regimens by patients and treatment protocols by prescribers [8].

Ghana became the 65th nation to join the WHO Programme for International Drug monitoring and started her pharmacovigilance and spontaneous ADR monitoring activities in 2001 [9]. Documentation of ADRs by health professionals is a major challenge of the program, where health professionals usually underreport cases of ADRs [10]. The WHO recommended a reporting rate of 200 cases per million population per year. A study conducted in Ghana found that for a standard of 100 cases per million people, a reporting rate of 23 was expected. However, only 1.55 was observed, despite the widespread availability of ADR forms in health facilities across the country [11].

Several studies conducted across the globe on pharmacovigilance have cited various reasons for underreporting of ADRs. While some studies reported unawareness of the reporting procedure, others reported ignorance of the reporting procedure as the common reason given by most health professionals [10, 12–15]. Since nurses spend more time with the patient on the ward and are likely to see patients developing ADRs, evaluating nurses' knowledge, practices, and barriers to pharmacovigilance and adverse drug reaction reporting can aid in the development of initiatives for enhancing reporting schemes to promote patient safety.

## Methods

### Study design

The study used a cross-sectional descriptive study that sought to assess the knowledge, practices, and barriers of pharmacovigilance among nurses. The study was conducted in a tertiary hospital in Ghana.

### Study participants and sample size

#### Inclusion criteria

The study participants were nurses with more than one year of clinical experience working in the teaching hospital's medical, surgical, or pediatric units.

#### Exclusion criteria

Student nurses and other health care workers were excluded from the study.

The sample size was calculated using the Krejcie and Morgan table to determine the sample size for the research activity [16].

### Sampling procedure

The total population of nurses in the hospital was 266. Due to the unequal distribution of nurses among the various nursing units, a stratified sampling technique was used to select 125 nurses to get a fairly representative study population. The investigators visited all the nursing units and folded papers with "YES" and "NO" inscriptions. The folded sheets were placed in a container and distributed to respondents who met the inclusion criteria. Those who selected "YES" during each visit were included in the study, and those who selected "NO" were not included. The response rate was 100%.

### Data collection procedure

The sampled population was given the participant information sheet with their permission, and explanations were provided where needed. The participant's right to participate at no cost or risk and to agree or disagree was emphasised. The questionnaire was self-administered to participants who willingly consented to participate in the research. Because all respondents were literate, the questionnaires were distributed to respondents, clarifications were made on items that respondents did not understand, and the investigators subsequently collected the questionnaires. Respondents spent an average of 30 min answering the questionnaires outright and returned the questionaire the same day. Those who could not complete the questionnaires on the same day were given 48 h to answer and return. All 125 questionnaires were retrieved, giving a response rate of 100%. Data were collected from October to November 2020.

### Data collection instrument

A structured questionnaire was developed and used for data collection based on the study's objectives and reviewed literature. The questionnaires were assessed for content and construct validity by two academic experts in pharmacovigilance and ADRs and measurement and evaluation. The questionnaire consisted of five sections. Part “A” covered the socio-demographic data of the respondents, and part "B" consisted of seven (7) knowledge questions with two options: "YES" for having knowledge and "NO" for not having knowledge of pharmacovigilance, and section "C" covered the purpose of reporting ADRs by nurses, which had six(6) items with two options: "YES" for knowing the purpose for reporting ADRs and "NO" for not knowing the purpose for reporting ADRs, section “D” comprised six questions that sought to find out barriers to reporting ADR.

The level of knowledge was measured using the total score of seven responses. Four (4) to seven (7) “YES” responses were graded high knowledge level, and one (1) to three (3) “YES” responses were graded low knowledge level. The purpose for reporting ADRs was measured using the total score of six responses. Three (3) to six (6) “YES” responses were graded as nurses knowing the purpose of reporting ADRs, and one (1) to three (2) “YES” responses were graded as nurses not knowing the purpose of reporting ADRs.

The reliability of the data collection instrument was checked and had an acceptable Cronbach’s α = 0.70 [17, 18]. The knowledge subscale of the data collection instrument had acceptable reliability, Cronbach’s α = 0.67. The purpose sub-scale the data collection instrument had acceptable reliability, Cronbach’s α = 0.87, and the barriers sub-scale the data collection instrument had acceptable reliability, Cronbach’s α = 0.67.

### Data analysis

The captured data were entered into the Statistical Package for Service Solution (SPSS) Version 25.0 database and analysed. Demographic characteristics, knowledge, purpose, practices, and barriers to pharmacovigilance among nurses were described using descriptive statistics. Analysing and interpreting the data were made easier by creating several relevant tables. Mean, and standard deviation was used to describe continuous variables with normal distribution. Categorical variables were described with frequencies and proportions in tables. A two-way between-groups analysis of variance (ANOVA) was used to determine the impact of nurses' rank and ward on pharmacovigilance practice. A correlation analysis was used to test the relationship between knowledge and practice of pharmacovigilance and the purpose of reporting ADR. A linear regression analysis was used to predict the influence of attending in-service training and practices of pharmacovigilance. A *p*-value < 0.05 was used to assess the level of significance at a 95% confidence level.

## Results

### Socio-demographic characteristics

The socio-demographic characteristics of the respondents covered the years of experience, sex, academic rank, and department in which respondents worked (Table 1). The mean age of the respondents was 33.17 years with a standard deviation of ± 6.465 years, and the mean years of working experience were 5.60 years with a standard deviation of ± 3.445 years. The majority (67.2%) of the respondents were females and 32.8% were males. Based on the education rank, most of the participants (37.6%) were staff nurses, followed by nursing officers (32.0%), and the educational rank least represented was principal nursing officers (1.2%).

**Table 1:** Socio-demographic characteristics of respondents

Biodata	Frequency	Percentage (%)
Age (years)	Mean (*SD) = 33.17 ± 6.465	
Working experience	Mean (*SD) = 5.60 ± 3.445	
Gender		
Male	41	32.8
Female	84	67.2
Total	125	100
Ward		
Medical	45	36
Surgical	42	33.6
Paediatric	38	30.4
Total	125	100.0
Educational qualification		
Staff Nurse (SN)	47	37.6
Nursing Officer (NO)	40	32.0
Senior nursing officer (SNO)	32	25.6
Principal nursing officer (PNO)	2	1.2
Deputy director of nursing service (DDNS)	4	3.2
**Total**	**125**	**100.0**

### Knowledge of pharmacovigilance among nurses

Out of the 125 nurses interviewed, 70.4% had heard of ADR reporting in Ghana, and 64.8% had not seen the form for reporting ADRs. Additionally, 68,8% of the respondents stated that they did not know the tools for reporting ADRs, and 71.2% did not know where to obtain the reporting tools for reporting ADRs in the hospital. Less than half (41.6%) of the study population had in-service training on drug safety and reporting ADRs. However, 75.2% did not know the information required on the ADR form. The level of knowledge was measured using the total score of seven responses. Four (4) to seven (7) yes responses were graded high knowledge level, and one (1) to three (3) yes responses were graded low knowledge level. The majority (71.2%) of the nurses had a low knowledge level of ADR reporting procedures (Table 2).

**Table 2:** Knowledge of nurses on ADR reporting procedures

Variables Sampling of participants	Frequency (%)	
	**YES**	**NO**
Have you heard about ADR reporting in Ghana	88 (70.4%)	37 (29.6%)
Have you ever seen the form for reporting ADRs	44 (35.2%)	81 (64.8%)
Have you had in-service training on drug safety and reporting ADRs?	52 (41.6%)	73 (58.4)
Do you know the tools used for reporting ADR in Ghana?	39 (31.2%)	86 (68.8%)
Do you know where to obtain the reporting tools for reporting ADRs in your hospital?	36 (28.8)	89 (71.2%)
Do you know the information that is required on the ADR form?	31 (24.8%)	94 (75.2%)
Do you know where to send the filled ADR form?	42 (32.8)	83 (67.2%)
**Level of knowledge of ADRs reporting procedures**	**Frequency**	**Percentage (%)**
High knowledge level	36	28.8
Low knowledge level	89	71.2

### The purpose of reporting ADRs

When respondents were asked about the purpose of reporting ADRs, the majority (84.8%) of the nurses knew the purpose of reporting ADRs. The benefits of ADR reporting as perceived by respondents are to identify safe drugs (80.8%), to calculate the incidence of ADRs (75.2%), to identify predisposing factors to ADRs (77.5%), to identify previously unrecognized ADRs (78.4%), and for comparison of ADRs of drugs within the same therapeutic class (77.6%). The purpose of reporting ADRs was measured using the total score of six responses. Three (3) to six (6) yes responses were graded as nurses knowing the purpose of reporting ADRs, and one (1) to three (2) yes responses were graded as nurses not knowing the purpose of reporting ADRs, as shown in Table 3.

**Table 3:** Purpose for reporting ADRs among nurses

Variables	Frequency (%)	
	**YES (%)**	**NO (%)**
To identify safe drugs	101 (80.8%)	24 (19.2%)
To calculate incidence of ADRs	94 (75.2%)	31 ((24.8%)
To identify predisposing factors to ADRs	97 (77.6%)	28 (22.4%)
To identify previously unrecognized ADRs	98 (78.4%)	27 (21.6%)
To serve as an information resource about the characteristics of the ADR	93 (74.4%)	32 (35.6%)
For comparison ADRs of drugs within the same therapeutic class	97 (77.6%)	28 (22.4%)
**The purpose of reporting ADRs among nurses**	**Frequency**	**Percentage**
Knowing the purpose for reporting ADRs among nurses	**106**	**84.8%**
Not knowing the purpose for reporting ADRs among nurses	**19**	**15.2%**

The bold mark up sums the overall score of nurses knowledge of the purpose of pharmacovigilance which was categorised as knowing the purpose for reporting ADRs among nurses and not knowing the purpose for reporting ADRs among nurses

### The practice of pharmacovigilance among nurses

Among the nurses interviewed, (47.2%) reported that they had nursed ADR cases in the last twelve months. The majority of the reported cases came from the medical ward (44.1%) followed by the surgical ward (35.6%) with the pediatric ward recording the least (20.3%) cases. It was also observed that female nurses nursed more patients with ADRs (69.5%) than their male counterparts (30.5%).

Nursing officers (NO) also nursed the highest number of patients with ADRs (39.0%), followed by senior nursing officers (SNO) (30.5%) and the principal nursing officers (PNO) and deputy director of nursing services (DDNS) who reported only (1.7%) nursed case respectively (Table 4).

**Table 4:** Practice of pharmacovigilance among nurses

Have you nursed a patient with an ADR in the past year?	Frequency (%)
Wards	
Medicine	26 (44.1%)
Surgery	21 (35.6%)
Paediatrics	12 (20.3%)
Total	59 (100%)
Gender	
Male	18 (30.5)
Female	41 (69.5%)
Total	59 (100%)
Ranks	
SN	16 (27.1%)
NO	23 (39.0%)
SNO	18 (30. 5%)
PNO	1 (1.7%)
DDNS	1 (1.7%)
**Total**	**59 (100%)**

Furthermore, among the nurses who reported having nursed a patient with ADRs, 52.5% stated that they reported the case, while (47.5%) did not report it. The following reasons were given by the nurses who did not report ADRs. The most common reason for not reporting ADRs was that the nurses thought the response was typical and usual with that drug (25%). Eight (28.5%) of the nurses stated that they were unaware that they were required to report. Furthermore, (28.5%) did not understand the reporting method, while (17.9%) claimed that the reporting forms were unavailable (Table 5).

**Table 5:** Reasons for not reporting ADRs among nurses

Reasons for not reporting ADRs	Frequency	Percentage (%)
I did not know I was supposed to report	8	28.5%
The reporting form was not available	5	17.9%
I do not know the reporting procedure	8	28.5%
I did not consider it important/serious	0	0.00%
I considered it “normal because it is a common reaction with that medicine	7	25%
To maintain clients’ confidentiality	0	0.00

### Two-way between groups analysis of variance of the impact of rank and ward of nurses on practices of pharmacovigilance

A two-way between-groups analysis of variance (ANOVA) was conducted to determine the impact of rank and ward of nurses on the practice of pharmacovigilance. There was no statistically significant impact of rank and ward of nurses on practices of pharmacovigilance (F(7,111) = 0.26, *p* = 0.968). Thus, the ward and rank of nurses did not significantly impact the practice of pharmacovigilance in the hospital (Table 6).

**Table 6:** Analysis of variance of the impact of rank and ward of nurses on practices of pharmacovigilance

Dependent Variable: Practice of pharmacovigilance						
Source	Type III Sum of Squares	df	Mean Square	F	Sig	Partial Eta Squared
Corrected Model	8.832 ^a^	13	.679	.962	.493	.101
Intercept	355.562	1	355.562	503.618	.000	.819
RANK	3.509	4	.877	1.242	.297	.043
WARD	2.048	2	1.024	1.450	.239	.025
RANK * WARD	1.289	7	.184	.261	.968	.016
Error	78.368	111	.706			
Total	1432.000	125				
Corrected Total	87.200	124				

^a^ R Squared = .101 (Adjusted R Squared = -.004)

### Relationship among knowledge, purpose, and practise of pharmacovigilance

The correlation analysis in Table (7) showed no correlation between knowledge and practice of pharmacovigilance and the purpose of reporting ADR. Knowledge of pharmacovigilance did not correlate with the purpose of reporting adverse drug reactions (r = 0.15, *p* = 0.099). Also, knowledge of pharmacovigilance did not correlate with the practice of pharmacovigilance (r = 0.02, *p* = 0.847). However, pharmacovigilance practices correlate negatively with the purpose of reporting ADR with r = -0.163, *p* = 0.069. This implies that as practices of pharmacovigilance increase, the purpose of reporting adverse drug reactions decreases. Thus, nurses knew the purpose of reporting ADR, yet they were not practicing it in the hospital.

**Table 7:** Correlation between knowledge, purpose, and practise of pharmacovigilance

Correlations				
		Knowledge	Purpose	Practice
Knowledge	Pearson Correlation	1	.148	.017
	Sig. (2-tailed)		.099	.847
	N	125	125	125
Purpose	Pearson Correlation	.148	1	-.163
	Sig. (2-tailed)	.099		.069
	N	125	125	125
Practice	Pearson Correlation	.017	-.163	1
	Sig. (2-tailed)	.847	.069	
	N	125	125	125

### Linear regression between attending in-service training and practice of pharmacovigilance

From the linear regression analysis, in-service training does not predict practices of pharmacovigilance, β = -0.095, *p* = 0.517 (Table 8).

**Table 8:** Linear regression between attending in-service training and practice of pharmacovigilance

Coefficients						
Model		Unstandardised Coefficients		Standardised Coefficients	t	Sig
		B	Std. Error	Beta		
1	(Constant)	3.321	.098		33.743	.000
	In-service training	-.095	.147	-.058	-.649	.517

### Barriers to the practice of pharmacovigilance

Nurses cited the following reasons as barriers to reporting ADRs: As shown in Table 6, lack of time and heavy workload (20%), unaware of the reporting procedure (20.8), fear that the report may be incorrect (16.0%), and lack of reporting form in hospitals (15.2%) were all factors (Table 9).

**Table 9:** Barriers to the practice of pharmacovigilance

Variables	Frequency	Percentage (%)
Concern that the report may be wrong	20	16.0%
Lack of time and heavy workload	25	20.0%
Unaware of the reporting procedure	26	20.8%
No idea that ADRs are to be reported	19	15.2%
The reporting form is not available in the hospitals	19	15.2%
Fear of being accused of wrongly administering a drug	16	12.8%
**Total**	**125**	**100**

## Discussion

The study was conducted to generally assess the knowledge, practices, and barriers to pharmacovigilance among nurses at a teaching hospital. Findings from this study showed that the majority (71.2%) of nurses did not have adequate knowledge of pharmacovigilance and their reporting system. It was evident that out of the 125 nurses, 64.8% had not seen the form for reporting ADRs. Less than half (41.6%) of the respondents had in-service training on drug safety and reporting ADRs, yet (75.2%) did not know the information that is required on the ADR form. Various educational platforms for healthcare professionals, such as pharmacovigilance training and workshops, are critical for increasing nurses’ knowledge, practices, and barriers concerning ADRs [19]. There was no statistically significant relationship between attending in-service training and reporting ADRs. Even though almost 42% of the respondents had some form of training on pharmacovigilance, their knowledge regarding pharmacovigilance was poor. The poor knowledge of pharmacovigilance could be attributed to the priority that this group of medical professionals place on the problem of ADRs, which is not promising. Additionally, another reason could be that the nurses who participated in the in-service training did not pay prompt attention during the training or that there were not sufficient learning materials because it is expected that those who attended the in-service training should know the required information on the ADR form. Since most of the nurses do not know the procedure for reporting ADRs, it will be difficult for them to report any adverse drug events. In contrast, in a study conducted in India, healthcare workers who received educational training on ADR reporting had a proper understanding of pharmacovigilance and better awareness of ADRs [20]. Healthcare workers should receive appropriate education and training regularly to improve their understanding of ADR reporting. Other research found that educational interventions improved ADR reporting awareness [21]. A study in Nepal showed that educational intervention might improve awareness of ADR reporting because many healthcare professionals have experienced ADR throughout their clinical practice but lacked adequate understanding of where and how to report ADR. Ongoing efforts are necessary to raise awareness of ADR reporting through the provision of education and training programs at regular intervals [22].

Findings from the study show that the nurses (84.8%) had good knowledge of the purpose of pharmacovigilance. This finding is supported by a study conducted in Karachi, Pakistan, where physicians had good knowledge of ADRs, but only 15.5 and 16% of them understood where and how to report ADRs, with only 7.5% of those surveyed having access to the ADR system [23]. Another study conducted in the Volta Regional Hospital of Ghana found that doctors, pharmacists, and nurse prescribers are knowledgeable about pharmacovigilance in Ghana [24].

Among the nurses interviewed, 47.2% indicated having nursed patients with an adverse drug reaction. When asked whether they reported the ADRs by filling out an ADR report form, 47.46% stated they did not report it. Findings from the current study on reporting ADRs are higher than the 42.5% found in the study carried out in Northern Nigeria [13], 41% found in medical practitioners in India [25], and 21% found among doctors in the Greater Accra Region of Ghana [10].

The most prevalent explanation for not reporting ADRs was that 28.5% of the nurses were unaware that they were required to report. Additionally, 28.5% did not know the reporting procedure, whereas 25% regarded the ADR as typical and usually linked with that drug. The results from the current study are similar to those conducted in Ibadan, Nigeria, among doctors, where unawareness of the presence of the ADR form and ignorance of the reporting procedure prevented reporting among doctors [12]. Another study conducted in Karachi, Pakistan, among physicians also reported that approximately 20% of the respondents considered that reporting a single ADR makes no significant contribution to the ADR reporting system, whereas 48% thought that ADR reporting generates extra work [23].

According to research conducted in Barcelona, a lack of time to report an ADR, the absence of an ADR reporting system in hospitals, and a lack of knowledge of the spontaneous reporting system were the primary causes of underreporting ADRs in Spain [26].

When the nurses were asked what would discourage them from reporting ADRs, the most common reasons stated were lack of time and heavy workload (20.0%), unawareness of the reporting procedure (20.08%), concern that the report may be wrong (16.0%), and unavailability of reporting from the hospitals (15.2%). Similar results have been observed in other studies. A study conducted among physicians in Ghana found that the biggest barrier to ADR reporting was a lack of time and a heavy workload. However, in that study, respondents reported a "lack of confidence in the reporting system" as a barrier to ADR reporting more frequently than "unavailability of the reporting form" [10].

Another study conducted in the United Arab Emirates also reported not knowing how to report, nonremuneration for reporting and lack of time to actively look for ADRs discouraged doctors from reporting ADRs [14].

### Limitation of the study

Because this is a cross-sectional study, it is impossible to draw definitive conclusions about the correlations between nurses' characteristics, knowledge variables, attitudinal variables, and practice variables. There is a possibility that respondents' recall and personal bias will influence the data and outcome of this study. No independent verification or authentication could be performed on the information provided about the number of ADRs that have ever been observed and reported. The study's findings are limited to teaching hospitals; therefore, they should be evaluated with those restrictions in mind.

## Conclusion

Nurses in this study had insufficient knowledge of pharmacovigilance and its reporting procedure but had good knowledge of the purpose of reporting ADR. Even though there was no statistical association between attending in-service training and practice of pharmacovigilance, hospitals should pay much attention to the in-service training of nurses, given the crucial role that nurses play in pharmacovigilance activities and adverse drug reaction reporting. Additionally, making the ADR reporting form more accessible, allowing for online submission of ADR reports, integrating electronic reporting, and giving encouragement and feedback can all help to improve ADR reporting performance in the long term.

## Data Availability

The availability of data and materials used in this study cannot be made public due to ethics concerns but are available from the corresponding author on reasonable request.

## Abbreviations

PV: Pharmacovigilance
ADRs: Adverse drug reactions
SADR: Spontaneous adverse drug reporting
SN: Staff nurse
NO: Nursing officer
SNO: Senior nursing officer
PNO: Principal nursing officer
DDNS: Deputy director of nursing service
